# Delirium management in perioperative geriatric services: a narrative review of non-pharmaceutical strategies

**DOI:** 10.3389/fpsyt.2024.1394583

**Published:** 2024-06-17

**Authors:** Rozenn Travers, Geoffroy Gagliardi, Maximilian Ramseyer

**Affiliations:** ^1^ Service de Court Séjour Gériatrique, Pôle Médecines Fortes Consultations, Centre Hospitalier Universitaire d’Orléans, Orléans, France; ^2^ Department of Neurology, Brigham and Women’s Hospital, Boston, MA, United States; ^3^ Department of Neurology, Massachusetts General Hospital, Boston, MA, United States; ^4^ Harvard Medical School, Boston, MA, United States

**Keywords:** case management, geriatrics, perioperative period, subacute delirium, non-pharmaceutical treatment

## Abstract

Delirium, a common complication in elderly surgical patients, poses significant challenges in perioperative care. Perioperative geriatric services (PGS) aim at managing comorbidities, postoperative complications, and initiating early recovery of mobility to enhance elderly patients’ prognosis in the perioperative period. Studies have shown that patients with preoperative cognitive disorders are at a significantly increased risk of postoperative delirium. While postoperative delirium affects up to 70% of people over 60 and 90% of people with neurodegenerative diseases, it remains underdiagnosed in many cases. Postoperative delirium can lead to functional decline, prolonged hospitalization, increased healthcare costs, cognitive impairment, and psychological malaise. This article briefly summarizes the literature on delirium, its risk factors, and its non-pharmaceutical management strategies within the perioperative period. It highlights the importance of integrating cognitive and psychological assessments into perioperative care protocols to provide baseline data, improve patient outcomes, reduce hospital stays, and minimize complications associated with delirium. By embracing evidence-based delirium management protocols, healthcare professionals can better identify and manage delirium, ultimately improving the quality of care for elderly surgical patients, which would also benefit healthcare staff and healthcare institutions.

## 1 Introduction

Elderly patients are considered to be at high risk of perioperative complications. Several factors contribute to the heightened occurrence of such complications, including cognitive impairment, the presence of neurodegenerative disease, depression, substance abuse, cardiorespiratory status, functional abilities, mobility and risk of falls, frailty, diet, medication intake, family and social support, and postoperative delirium (1). Postoperative delirium has been reported in up to 70% of people over 60 years of age (2) and up to 90% of people with neurodegenerative diseases (3). These states of post-surgical confusion remain underdiagnosed for an estimated 40% to 60% of hospitalized patients and for patients with pre-existing neurodegenerative disease the estimates indicate a misidentification rate as high as 88% (3).

The aims of perioperative geriatric services (PGS) include managing comorbidities, postoperative complications, and initiating early recovery of mobility (4–6) to improve patient prognosis, e.g. by reducing both in and out-of-hospital mortality at 6 months (5).

## 2 Delirium

### 2.1 Definition

Various labels refer to syndromes of confusion, including confusion, confusional state, acute confusional syndrome, and mental confusion. The Diagnostic and Statistical Manual of Mental Disorders-V uses the term delirium (7). The criteria used to define delirium include impairments in attention, vigilance, and cognition, i.e. memory, language, perceptual disorders, disorientation, that cannot be attributed to a pre-existing neurological disease. This impairment occurs within a few hours or days, fluctuates during the day and cannot be explained by other medical issues, intoxications, withdrawal symptoms, use of drugs or any other cause.

Similarly, the International Classification of Diseases (Eleventh Revision, ICD-11; 8) diagnoses delirium based on changes from baseline functioning over a short time window, i.e., hours to days, that cannot be explained by pre-existing or evolving neurocognitive or mental disorders. However, this classification focuses on more restricted cognitive domains, i.e., attention, orientation and awareness. This definition also accounts for external causes that may lead to delirium, including psychoactive substances and other medical conditions. These results suggest that positive patient outcomes depend upon promptly diagnosing and managing delirium and its triggers (3).

### 2.2 Types of delirium

Delirium can be characterized by its duration or by its behavioral phenotype. Behaviorally, delirium presents in three forms: hypoactive, hyperactive and mixed.

Hypoactive delirium, constituting approximately 25–50% of cases (9–11), is characterized by passivity, introversion, psychomotor slowing, decreased alertness and communication (2, 12–14). This phenotype resembles severe psychoaffective syndromes, i.e. depression or anxiety, complicating its diagnosis (15, 16). Accurate diagnostic differentiation is crucial as treatment varies between these syndromes. (13, 16).

In contrast, hyperactive delirium is marked by motor agitation, aggression, hallucinations and delusions (2, 12–14). Hyperactive confusion, comprising 21.5% to 25% of confusional states, (9–11), is marked by heightened behavioral disturbances, distinguishing it from dementia syndromes (11, 16).

Finally, mixed delirium, encompassing approximately 25% of confusional syndromes (9), combines the two previous states with rapid fluctuations, i.e. over less than 24 hours (2).

Delirium is frequently mistaken for dementia, depression or chronic hallucinatory psychosis, with an estimated 50% of cases remaining undiagnosed (15), thus extending its clinical duration (9).

Previous studies have found that delirium is associated with a one year increased risk of mortality compared to patients without delirium (17). Subsequent authors investigated the duration and survival rates associated with differing types of delirium. Van Den Boogaard and colleagues found that each delirium subtype has significantly different durations, i.e., the hyperactive (median: 1, IQR: 1–1) and hypoactive (median: 1, IQR: 1–4) subtypes’ median duration is one day while the mixed subtype can last up to four days (IQR: 2–13; 18). Multiple studies converge on the finding that the hyperactive subtype has both the lowest duration and lowest associated mortality risk (10, 19). By contrast, the hypoactive and hypoactive with mixed features syndromes were associated with significantly higher mortality risks. Furthermore, Yang and colleagues showed that the mortality rates significantly depended on the presence or absence of dementia. In cases when dementia was absent, the severity of the disease (10).

### 2.3 Risk and protective factors

Numerous risk factors predisposing individuals to delirium are known. These include factors related to the patient’s idiosyncrasies, such as age (9, 16, 20, 21), mobility (15), perceptual abilities (9, 15, 16) or sleep deprivation (15). Health status, especially comorbidities (2, 15, 22), also plays a significant role along with metabolic disorders (15, 21, 23), psychiatric disorders (9, 11, 16, 20, 24, 25), addiction (16), cognitive impairment or neurodegenerative diseases (9, 15, 16, 21), and even the functional status in general (21). Postoperative complications, e.g. infections, fever, dehydration, can also influence delirium onset (2, 15, 22).

Among these factors, age seems to be a significant risk factor. Studies report the proportion of hospitalized patients experiencing delirium may increase as a function of age (15). While up to 48% of patients aged of 65 years and older are diagnosed with delirium (26), the proportion increases to 56% for patients above 80 years of age (15). Studies have also found different incidences of delirium depending on the type of surgery. Cardiac surgery results in postoperative delirium in 23% to 73% of cases, whereas orthopedic surgery causes delirium in 28% to 52% of cases. Overall, surgery may lead to delirium onset between 23% (27, 28) and up to 73% of the time (20) depending on the sample. Some authors have also shown that between 17% to 39% of delirium symptoms might stem from iatrogenic phenomena, making medication one of the most important potential risk factors (15). Psychiatric symptoms also constitute an another important preoperative risk factor, as depression affects 12% (25) to 23% (27) of the observed sample and may increase the risk of delirium onset by 21% (27). Lastly, patients’ preoperative cognitive state is a strong risk factor, as some studies report that 90% of people with neurodegenerative diseases may exhibit delirium (3).

Patients are at a high risk of suffering from delirium if they display at least three of the above factors (22) or if they are subject to certain conditions, e.g. recent orthopedic surgery, vascular or cardiac complications (13). Prompt management of these factors and symptoms is crucial, affecting both hospital stay length and patient outcomes (2). Yet, in about 25% of cases, delirium may arise without medical causes (3), potentially due to inadequate care or stress related to an elderly person’s inherent frailty.

Protective factors can reduce the frequency and delirium-related complications (12). In the preoperative phase, this involves monitoring hydration, pain, oximetry, medication and nutrition (29). Addressing these protective factors either helped reduce the severity or duration of delirium (29, 30), or diminished memory impairment (30). Authors estimate that these protective factors may diminish the risk of delirium onset by 40% (15). For example, Inouye and colleagues showed that by controlling for six different major factors, i.e., cognition, sleep, mobility, vision, audition, and hydration, delirium incidences and delirium duration were significantly reduced from 15.5% to 9.9% and from 161 to 105 days, respectively (26).

Physical activity (2), intellectual activity (31), and cognitive reserve (2, 31) are additional important factors that modulate brain plasticity (32–34). Past studies have shown that surgery-related anesthesia is associated with increased risk of neurodegenerative diseases such as Alzheimer’s (35) and Parkinson’s (36) diseases. However, the authors suggest that these procedures are not themselves the cause of cognitive impairment or neurodegenerative conditions (22), but may rather trigger cognitive reserve’s decline (37) and expose pre-existing neurological damage that was formerly compensated by cognitive reserve. Therefore, the later onset of such postoperative neurocognitive disorders is more likely revealed by the surgery rather than caused by it. Cognitive reserve appears to serve as a protective factor, as some studies have shown that higher reserve is associated with lower risk of postoperative delirium onset. This is especially the case for women as up to 38% of women’s delirium risk is reduced with each +0.5SD in a standardized reading test used as a proxy for cognitive reserve (28).

### 2.4 Scales for delirium examinations

The most widely used delirium scale for elderly patients (2) is the Confusion Assessment Method (CAM; 38). This tool is widely used and has been translated into several languages, including Spanish (39), French (40), German (41), Italian (42), Japanese (43), Chinese (44), etc. It assesses four areas: 1) Acute onset and fluctuating course, 2) Inattention, 3) Disorganized Thinking and 4) Altered Level of Consciousness (38, 40). It is also applicable for depression and neurodegenerative disease (38, 40) and its administration requires approximately five minutes for a trained examiner (3, 15, 22, 38). The scale demonstrates high sensitivity, i.e. 94–100%, specificity, i.e. 89–95%, (2, 20, 22) and satisfactory inter-examiner reliability, i.e. between 0.81 and 1.00 (38).

The Delirium Symptom Interview (DSI; 45) is a diagnostic measure designed for daily administration, which accounts for delirium’s potential delayed onset and symptoms’ fluctuation over time. The questionnaire, grounded in DSM-III delirium criteria, targets specific symptoms and takes 10–15 minutes to complete. It has shown high sensitivity and specificity, at 90% and 80%, respectively. (45).

Predicated on DSM-IV criteria, the Memorial Delirium Assessment Scale (MDAS; 46) is a 10-minute scale that assesses delirium severity. It measures ten characteristics, including patients’ level of consciousness, cognition, perceptual disturbances and delusions, psychomotor activity and sleep-wake cycle disturbances. It can be administered several times a day, offering clearer insights into the delirium type, i.e. hypoactive vs. hyperactive.

## 3 Preoperative psychological and cognitive assessments

Given the heightened risk of postoperative delirium in patients with cognitive disorders, severe disease or visual impairment, a pre-surgical cognitive screening (1, 47, 48) or a comprehensive neuropsychological assessment is recommended (47). This evaluation informs on the patient’s prior cognitive condition (49) and guides post-surgery care adjustments (50).

### 3.1 Cognitive assessment

Screening tests are usually based on the Mini Cog test (51), the COgnitive Disorder EXamination (CODEX; 52), the Montreal Cognitive Assessment (MoCA; 53), or the Mini Mental State Examination (MMSE; 54).

If cognitive issues are suspected after screening, a more detailed neuropsychological assessment is required (48, 50). This evaluation establishes a baseline (16, 49), aiding in planning care and preventive actions to lower the risk of autonomy loss, cognitive disorders, or mental confusion onset. (47, 50). Preoperative cognitive disorders significantly increase the duration of postoperative delirium (55). However, delirium onset may begin before the operation for some patients. Delirium can stem from pain, bone fracture-related neurovascular damage, analgesics, etc. (56).

Interviewing patients’ family and friends can yield insights into the cognitive difficulties’ characteristics, onset, progression, and effects on daily activities. This information is also useful for detecting pre-operative confusion and provides an overview of patients’ everyday cognitive and functional dysfunctions.

### 3.2 Psychological assessment

Studies have shown that preoperative depressive symptoms, i.e., 48 hours prior to surgery, was significantly associated with delirium’s onset, duration, and increased pain perception (25, 57). Screening for depression is recommended to determine patients’ treatment plan and to optimize postoperative outcomes (57). In these cases, several self-report questionnaires are typically used, including the Geriatric Depression Scale (GDS; 58, 59), which demonstrates high accuracy in diagnosing major depression, i.e., 84% specificity and 95% sensitivity for cut-off scores at 11/30 (60). GDS is often used in both clinical and research settings and also has a shorter version available (58). The Beck Depression Inventory (BDI; 61), the Hamilton Depression Rating Scale (HDS; 62, 63) and the Montgomery Asberg Depression Rating Scale (MADRS-S; 64) are other efficient and frequently used tools to measure depression, i.e., 86% sensitivity and 64% specificity for the BDI; 85% and 69% for the HDS; 85% and 79% for the MADRS; and 74% and 80% for the GDS 15-items (65). Although these tests and others are widely used to diagnose major depression, to our knowledge, there is no precise data about their performance in the specific context of delirium.

## 4 Postoperative delirium’s burden on the elderly

### 4.1 Functional burdens

Postoperative delirium can lead to numerous burdens for patients. The onset of delirium may be associated with functional decline, including loss of autonomy (17, 30, 66–68) and an increased risk of subsequent institutionalization (11, 55, 69). Medically, it may exacerbate dementia syndrome and raise mortality risk (11, 40, 55).

### 4.2 Financial burdens

The onset of delirium entails significant total healthcare costs for both institutions and families alike. Overall, delirium involves longer hospital stays (40, 55), i.e., up 7 to 11 more days (24, 68), and increased care requirements, implying higher costs for the healthcare system (9).

Comparisons between multiple studies have found that a patient with delirium costs 1.5 (70) to 2.5 (71) times more than a patient without delirium. Specifically, additional costs directly attributable to the presence of delirium was estimated to be approximately $44,291 per patient per year (70). In total, additional costs associated with delirium per patient and per year varies between studies and from one country to another. In the United States of America alone, studies estimate the additional cost of delirium is between $60,516 and $64,421 per patient per year (71), resulting in additional health care costs between $32.9 billion to $152 billion a year depending on different estimates (71, 72). Authors also note that the majority of costs attributable to delirium stems from the first 90 days of hospitalization (70).

Delirium management involves different treatments and requirements, each of which has different costs. When it comes to disease management and healthcare, authors distinguish between different types of costs, i.e., direct and indirect costs. Studies estimate that the total cost breakdown for delirium treatment management is 60% for personnel costs, 30% for medical services, and 10% for medication (73).

Direct costs consist of both healthcare, i.e., costs related to diagnosis, treatment, rehabilitation, and materials related to medical care expenditure, and non-healthcare costs, e.g., costs related to transportation, relocation, household expenditures and items related to consumption of non-healthcare resources in general (74). Healthcare costs include the purchase of pharmaceutical materials, i.e., antibiotics, intravenous fluid, and drugs. Studies have shown that the cost of pharmaceuticals accounts for the higher proportion of the total expenditures (73). Authors estimate that some additional costs, such as institutionalization, rehospitalization and stays for rehabilitation after the acute phase, nursing home use, and home healthcare would also be included within delirium’s direct costs (12, 70, 75) and would represent approximately $50,927 per patient per year (70). Notably, some studies have found that the direct costs were indexed according to the severity of the syndrome, i.e., $83,534 for patients with low to moderate delirium, $99,756 for moderate delirium, and $140,008 for severe delirium (70).

Indirect costs consists of delirium’s downstream consequences, including additional time taken from the healthcare team, which can be due to increased morbidity, mortality or impairment, or even additional time spent with the family and/or visitors (74). Weinrebe and colleagues estimate that the amount of additional time required of healthcare personnel depends on the type of delirium. Patients with prevalent delirium necessitate approximately 260 additional minutes of care and treatment whereas patients with incidental delirium need approximately 215 additional minutes (73). In the USA, indirect costs stemming from longer hospital stays associated with delirium account for an approximate $6.9 billion annual expense increase (12).

Leslie and colleagues estimate that the implementation of interventions proven to be effective to manage and reduce delirium might reduce the total costs attributed to delirium by $30 to 35 billion within a year (71). For example, the cost of testing cognition, i.e. MMSE, and delirium, i.e. CAM, in hospitalized elderly people would be significantly lower than the estimated cost of managing and treating mental confusion. These tests would therefore prevent subsequent procedures and expenses in approximately 50% of cases (75). Additionally, Inouye and colleagues estimate that using preventive measures would prevent both direct, e.g., medication, rehabilitation, and indirect, i.e., healthcare team time, costs and savings expenses by reducing 25% of delirium incidences (75). Studies have shown that the HELP program, with operating costs ranging from $86,886 to $309,172 including personnel and supplies, yields an annual financial return between $1,007,474 and $6,204,336. This equates to a return per patient of $386 to $532 (76). In a nursing home, the implementation of the HELP program saved $9,446 per patient per year (77).

Aside from direct and indirect costs, delirium also involves costs for third-party payers, the patients and their families (74). These can involve expenditures such as transportation, household, relocation, as well as direct consequences from informal care provided to their relatives. Although the overall cost of these consequences is difficult to estimate, the reduction of the incidence of delirium as well as its severity can significantly reduce expenditures.

### 4.3 Cognitive burdens

Delirium is associated with the onset (12, 78) and sometimes persistence of cognitive disturbances over several months or years (12, 49, 79), irrespective of preexisting cognitive impairment (40). “Postoperative cognitive dysfunction” (POCD) refers to such deterioration, which is temporally linked to the operation and observed in subsequent weeks or months (79). The sudden onset of cognitive impairment, measured by comparing preoperative and postoperative MMSE scores, may lead to delirium and neuropsychological disturbances (15). The latter may persist in 45.1% of elderly patients one month after surgery (69). For example, Krogseth et al. (67) tracked the six-month progression of delirium in elderly patients without neurodegenerative diseases following hip surgery. Almost a third of the participants assessed by CAM exhibited acute-phase delirium, and some individuals’ POCD continued to persist thereafter. Six months later, 15% of the sample was diagnosed with a neurodegenerative pathology, the majority of whom had experienced postoperative delirium. The authors suggest that developing delirium in the acute phase strongly predicts a diagnosis of dementia 6 months later.

When patients with delirium require postoperative assessment, the authors suggest initially delaying this examination (15, 22). They argue that an assessment during the acute phase cannot provide reliable and stable information on patients’ present or future cognitive state. Although there is no consensus on the time frame, some authors recommend delaying assessment for three to six months (15, 22).

### 4.4 Psychological burdens

Since preoperative depression is correlated with poorer prognosis for recovery time, functional remission, and longer hospital stays, it should be detected and managed as early as possible (J. 25).

Postoperative psychological risks must also be taken into account, particularly the post-fall risk (4) and depression (47). The latter is sometimes difficult to detect, as the emotional blunting that stems from depression can be mistaken for hypoactive delirium symptoms. Patients with depressive symptoms are known to report higher perceptions of pain and increased use of postoperative analgesics (80, 81).

Post-fall syndrome might necessitate psychological support to address activity limitations from the fear of falling, PTSD onset, or negative thoughts due to lost self-esteem and fear of death.

In the event of delirium onset, it is important for patients to be treated within a calm, reassuring setting. It is recommended to keep patients within a familiar environment, e.g., to avoid staff and room changes, to display familiar objects, to encourage communication between patients and their social surrounds, i.e., with nursing staff and family, and to recruit patients’ personal support systems to provide them with moral support (12).

## 5 Prevention of Postoperative Delirium through non-pharmacological measures

### 5.1 Healthcare staff training

The American Geriatrics Society Expert Panel on Postoperative Delirium in Older Adults recommends training healthcare professionals to identify, treat, and manage delirium (14). Such training can cover etiology, risk factors, assessment/screening, prevention, and disease management, i.e. pharmaceutical and non-pharmaceutical. Lundström et al., (82) proposed a four step program: 1) “A 2-day course for staff on geriatric medicine focusing on assessment, prevention, and treatment of delirium”, 2) “Education concerning caregiver-patient interaction focusing on patients with dementia and delirium”, 3) “Reorganization from a task-allocation care system to a patient-allocation system with individualized care”, 4) “Guidance for nursing staff once a month”. These training sessions enable care teams to be more attentive to the risk factors for the onset of delirium (cf. I.3) and prevent it through psychological support, interaction, non-pharmacological stimulation, spatio-temporal reorientation, hydration and sleep monitoring, mobilization, sensory stimulation, e.g., ensuring the patient wears glasses and/or hearing aids (5, 12, 15). Such training and management may reduce the risk of mental confusion, length of hospitalization (15, 82) and mortality (82) for patients experiencing delirium.

### 5.2 Geriatricians’ recommendations

In a study of patients undergoing hip fracture surgery, researchers showed that a preoperative geriatric consultation led to a one-third reduction in the occurrence and incidence of delirium compared to standard care, i.e., 32% vs. 50% of patients with postoperative delirium, respectively (29). During the interview, geriatricians provided recommendations on oxygen supply, pain management, hydration, drug management, nutrition, early mobilization and rehabilitation, detection, prevention, and treatment of postoperative complications, and postoperative environmental stimulation.

### 5.3 Cognitive stimulation

To explore the potential benefits of cognitive stimulation on the incidence and severity of delirium in elderly pre-surgery patients, Tow et al., (31) offered their patients activities such as reading the newspaper, knitting, card games, computer games, crosswords, bingo, singing, writing etc. They discovered that increased participation in these activities led to a lower incidence and severity of delirium. This was true for patients both with mild cognitive impairment and no cognitive impairment. The study emphasizes cognitive reserve and lifelong intellectual stimulation’s roles in mitigating postoperative mental confusion.

### 5.4 The Hospital Elder Life Program (HELP)

Some authors advocate for the use of non-pharmacological strategies as the first line of treatment for delirium (22). The aim of these strategies is to re-establish contact between the patient and reality. Inouye and colleagues (83) developed the Hospital Elder Life Program (HELP) to address the various factors associated with the emergence of postoperative delirium. This program focuses on six categories of risk factors: 1) cognition, 2) sleep, 3) mobility, 4) vision, 5) hearing, and 6) hydration. Specific stimulation is provided for each, e.g., reminding patients of caregivers’ names and spatiotemporal reorientation for cognition; respecting sleep-wake rhythms, relaxing music and massage for sleep; external compensation devices for vision, etc. (16, 22).

As the most common strategy (2), the HELP program has shown that monitoring these six factors can reduce delirium from 15% to 9% for patients with usual care (26). HELP has also shown to reduce the duration of delirium from 161 to 105 days (26) as well as its associated institutional costs (22, 75).

While this method effectively prevents the onset of delirium, its efficacy is limited once delirium is already established (75).

## 6 Conclusion

Appropriate management of preoperative and postoperative delirium appears to be of prime importance for patients, as it influences their short and long-term prognosis. Its assessment and management should begin as soon as patients are admitted (56). Prior evidence indicates that non-pharmacological interventions not only reduce delirium’s duration and healthcare cost for patients, families, and public health institutions, they also reduce the risk of negative functional and medical outcomes, i.e. institutionalization, medical complications, onset of cognitive impairment and dementia. Implementing non-pharmacological strategies with or without medication is feasible in hospital settings, offering benefits for patients, healthcare staff, e.g., reduced workload, and hospital administration, i.e., cost efficiency. This review highlights the importance of integrating cognitive and psychological assessments into perioperative care protocols for delirium prevention and management, with the aim of ultimately improving the quality of care for elderly surgical patients.

## Author contributions

RT: Conceptualization, Writing – original draft, Writing – review & editing. GG: Writing – original draft, Writing – review & editing. MR: Writing – original draft, Writing – review & editing.
